# Magnetite-Based Biosensors and Molecular Logic Gates: From Magnetite Synthesis to Application

**DOI:** 10.3390/bios13030304

**Published:** 2023-02-21

**Authors:** Nataliia Dudchenko, Shweta Pawar, Ilana Perelshtein, Dror Fixler

**Affiliations:** 1 Bar-Ilan Institute of Nanotechnology & Advanced Materials (BINA), Bar Ilan University, Ramat Gan 5290002, Israel; 2 Faculty of Engineering and Bar-Ilan Institute of Nanotechnology & Advanced Materials (BINA), Bar Ilan University, Ramat Gan 5290002, Israel

**Keywords:** magnetite nanoparticles (MNPs), surface functionalization, biosensors, point-of-care testing, molecular logic

## Abstract

In the last few decades, point-of-care (POC) sensors have become increasingly important in the detection of various targets for the early diagnostics and treatment of diseases. Diverse nanomaterials are used as building blocks for the development of smart biosensors and magnetite nanoparticles (MNPs) are among them. The intrinsic properties of MNPs, such as their large surface area, chemical stability, ease of functionalization, high saturation magnetization, and more, mean they have great potential for use in biosensors. Moreover, the unique characteristics of MNPs, such as their response to external magnetic fields, allow them to be easily manipulated (concentrated and redispersed) in fluidic media. As they are functionalized with biomolecules, MNPs bear high sensitivity and selectivity towards the detection of target biomolecules, which means they are advantageous in biosensor development and lead to a more sensitive, rapid, and accurate identification and quantification of target analytes. Due to the abovementioned properties of functionalized MNPs and their unique magnetic characteristics, they could be employed in the creation of new POC devices, molecular logic gates, and new biomolecular-based biocomputing interfaces, which would build on new ideas and principles. The current review outlines the synthesis, surface coverage, and functionalization of MNPs, as well as recent advancements in magnetite-based biosensors for POC diagnostics and some perspectives in molecular logic, and it also contains some of our own results regarding the topic, which include synthetic MNPs, their application for sample preparation, and the design of fluorescent-based molecular logic gates.

## 1. Introduction

In recent years, synthetic magnetite nanoparticles (MNPs) have been of great interest in a diverse number of applications, ranging from pigments to advanced molecular logic devices. Among the applications of MNPs are biomedical applications, such as in magnetic drug targeting [1,2], as contrast agents for MRI [3], and in magnetofection [4,5], hyperthermia [6], DNA/RNA purification [7,8], tissue engineering [9], theranostic platforms [10,11], smart biosensors [12,13], etc. They also have environmental applications, such as in purifying water [14], soil [15], and air [16] from heavy metals and other industrial pollutions; and optical applications [17], such as photonic materials [18], organic light-emitting diodes (OLEDs) [19], magnetic field sensors [20], “smart” windows [21], and solar energy harvesting [22].

Significant efforts are being made regarding the development of smart biosensors for real-time detection, and their importance is hard to overestimate. Currently, such smart biosensors are widely used in various fields, including food safety [23,24], environmental monitoring [25,26], clinical diagnostics [27,28,29], and treatment [30,31], and even in the detection of biological weapons [32,33]. Briefly, biosensing technology could be described as follows: a target analyte reacts with a recognition system equipped with a suitable transducer, which converts a biosensing event (physical, chemical, or biological) into measurable parameters [34]. In addition, sensing technologies (transducers) are utilized in biosensing: electrochemical [35], optical [36], photoluminescent [37], mass-sensitive [38], thermal [39], magnetic [40,41], etc. Among other sensors, the main features of POC sensors are their sensitivity and ability to achieve rapid diagnostics on the spot. Here, we review all the existing magnetite-based biosensing technologies, among a myriad of other sensors, as magnetite has particular advantages in biosensing [42]: (1) magnetite synthesis is relatively inexpensive; (2) covered and functionalized MNPs have high chemical and colloidal stability; (3) unique magnetic properties allow for the manipulation of nanoparticles by an external magnetic field; (4) the background signal of the biosensors is low (biological samples usually do not possess noticeable magnetic signal).

Magnetite, Fe_3_O_4_, is an iron oxide with a spinel structure composed of iron ions, Fe^3+^ and Fe^2+^, within the stoichiometry Fe^2+^/Fe^3+^ = 0.5. At room temperature, it is ferrimagnetic, with rather high saturation magnetization (~90 A·m^2^/kg). Such saturation magnetization makes it possible to easily manipulate MNPs with an external magnetic field. The Curie temperature of pure stoichiometric magnetite is around 580 °C. To date, various strategies for MNP synthesis have been developed, in which two main approaches have been used: top-down and bottom-up [43]. Top-down approaches involve physical methods and are based on crushing macroscopic pieces of material with the formation of nanoparticles, while bottom-up approaches include both chemical and biological methods and involve nanoparticles’ formation using molecules and ions. The surface of synthesized MNPs should be properly functionalized with various biomolecules for their utilization in magnetite-based biosensor development. These biomolecules (nuclear acids, proteins, lipids, etc.) form a “corona” on the surface of MNPs, which confers biological attributes to the nanoparticles [44].

This review provides a comprehensive overview of MNP synthesis and functionalization and focuses on recent advancements in magnetite-based biosensors for POC diagnostics and some perspectives from molecular logic. Firstly, MNP synthesis approaches are briefly described, as well as various methods of MNP surface coverage and functionalization for biosensing applications (Section 2). Then, the application of MNPs for diagnostic purposes, including biosensor development as well as POC diagnostics, is highlighted (Section 3). Finally, some perspectives on the use of MNPs for molecular logic are described (Section 4). Challenges in MNPs’ utilization for biosensing and molecular logic, as well as some future perspectives, are discussed (Conclusions). This review not only promotes recent methods of MNP synthesis and functionalization but also discusses novel cutting-edge MNP applications for POC sensors and molecular logic.

## 2. MNP Synthesis, Surface Coating, and Functionalization

As mentioned above, there are two main routes for the synthesis of MNPs: top-down and bottom-up. The top-down route includes physical methods, whereas bottom-up routes are based on chemical and biological methods. It is well known that uncoated synthesized MNPs are unstable in aqueous media and are easily precipitated with aggregate formation due to their high magnetization, so further coating of such MNPs is necessary to widen the scope of their application. After that, the functionalization of synthesized MNPs with biomolecules is an obligatory step in the production of biosensors, as it leads to the formation of active groups of MNPs on the surface (recognition system), which would react with the target biological object. The proper coating and functionalization of MNPs with shells and functional macromolecules affect the successful design of biosensors.

### 2.1. MNP Synthesis

**Figure 1:**
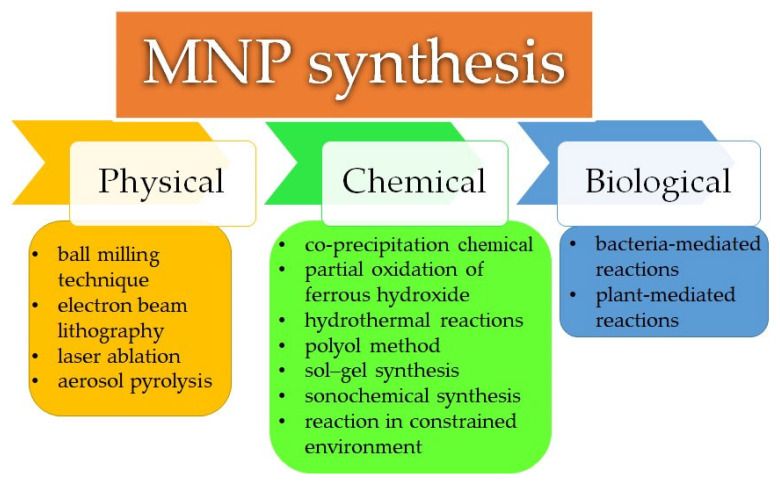
The main routes of MNP synthesis.

Despite the huge number of scientific publications devoted to MNP synthesis, here, we briefly discuss the main options (Figure 1).

#### 2.1.1. Top-Down Route (Physical)

Physical approaches to MNP synthesis include the methods of ball milling, electron beam lithography, laser ablation, pyrolysis, etc.

The ball milling technique [45,46,47] was developed in the 1970s and involves crushing bulk material in a hollow cylindrical jar with steel balls. It is a rather simple technique and is convenient for large-scale production, but it has the following disadvantages: the sizes and shapes of the obtained nanoparticles vary significantly, and the product is usually contaminated.

Electron beam lithography exploits electron beams (e-beams) for the transformation of iron into magnetite [48,49]. This method is based on the evaporation of iron in a vacuum with its further condensation in various matrixes (organic/inorganic) and the subsequent oxidation of iron nanoparticles to magnetite in the air. The main disadvantages of this method are that it is expensive and requires highly complex equipment, which needs a large amount of energy for nanoparticle production.

The laser ablation method utilizes lasers for the evaporation of surface atoms from a solid bulk target material in both gas and liquid phases. The subsequent nucleation and agglomeration of primarily formed clusters lead to nanoparticle formation [50]. The main challenge in MNP synthesis using laser ablation is the selective formation of an iron oxide phase [51]. Among the advantages of this method are its simplicity and cleanness without the need for extreme environmental conditions (pressure and temperature) [52].

MNPs could also be produced using the method of aerosol pyrolysis under a controlled atmosphere [53]. To obtain MNPs in the iron oxidation state, the fuel/air/inert gas ratios need to be controlled. The result also depends on the valence state of the iron precursors.

A brief summary of these physical methods of MNP synthesis is that they are generally complex, mostly utilize highly expensive equipment, and lead to the formation of various phase contaminations.

#### 2.1.2. Bottom-Up Routes (Chemical and Biological)

Chemical and biological approaches to MNP synthesis allow better control the shape and morphology of the synthesized particles, as well as the phase composition, to be controlled more effectively. Among these approaches are the following methods: co-precipitation, the partial oxidation of ferrous hydroxide, hydrothermal reactions, the polyol method, sol–gel synthesis, sonochemical synthesis, reactions in constrained environments, bacteria-mediated reactions, and plant-mediated reactions [54].

The most commonly used method to obtain MNPs is the co-precipitation method, which was first reported by Massart in 1981 [55]. Briefly, using this method, ferrous and ferric salts are co-precipitated in a water medium with a strong base solution to form MNPs [56]. Various factors could influence the properties (size, shape/morphology, magnetization, etc.) of the synthesized MNPs; among them are the ferrous and ferric salt concentrations, the Fe^+3^/Fe^2+^ ratios, the concentration and nature of the base solution, the temperatures of the reaction (room temperature or elevated), the stirring rate, the inert atmosphere, etc. This method is simple and could be easily scaled-up; however, it is hard to control the shape of the synthesized MNPs (poor morphology) and non-stoichiometric magnetite could be formed using this reaction.

**Figure 2:**
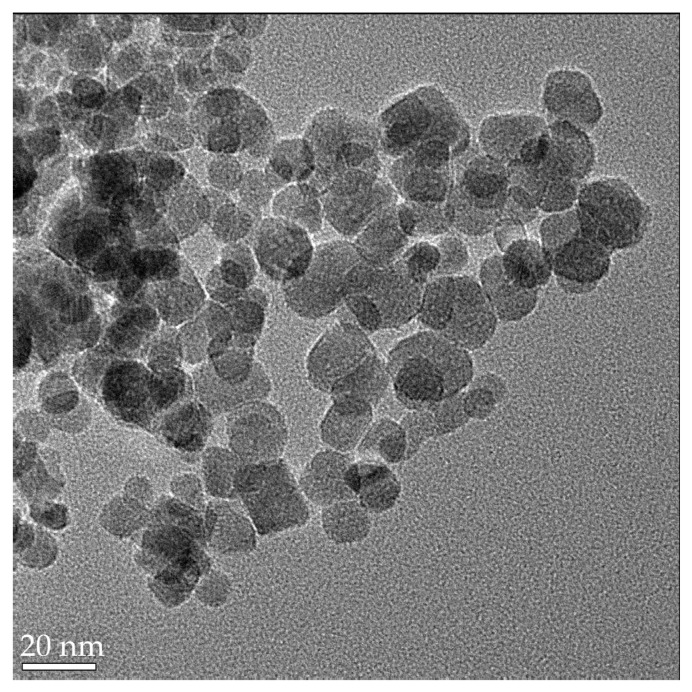
A TEM image of MNPs synthesized via the co-precipitation method (the results obtained in the laboratory of the presenting authors).

A TEM image of magnetite nanoparticles synthesized using the co-precipitation method is shown in Figure 2. Magnetite nanoparticles were synthesized via the co-precipitation of ferric and ferrous chlorides (at the Fe^+3^/Fe^2+^ ratio 2:1) with ammonia hydroxide and further incubation of the mixture at 90 °C for 30 min. The obtained nanoparticles were washed with water and separated magnetically.

The next widely used method used to obtain MNPs is the partial oxidation of ferrous hydroxide, which was first reported by Sugimoto and Matijevic in 1980 [57]. According to this method, in the first stage, amorphous ferrous hydroxide is precipitated from a ferrous sulfate solution with subsequent aqueous gel aging at 90 °C in the presence of a nitrate ion, which results in magnetite formation. Using this method, one could obtain “monodispersed” hydrophilic spherical MNPs of various sizes. The following reaction conditions define the properties of synthesized MNPs: reagent concentration, the availability of oxygen in the reaction mixture, the pH value of the reaction mixture, etc. This method is convenient; however, minor amounts of oxygen in the system lead to goethite formation, and the synthesized MNPs are extensively agglomerated.

**Figure 3:**
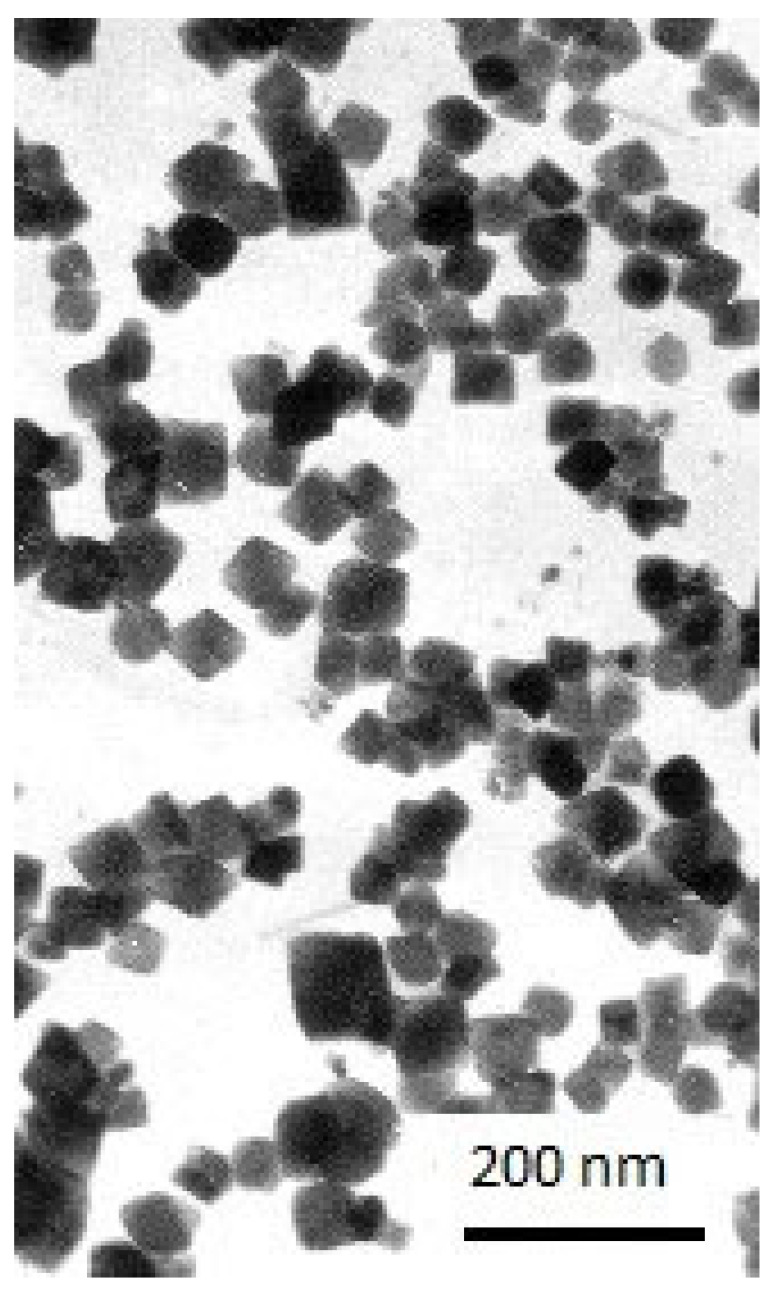
A TEM-image of MNPs synthesized via the partial oxidation of ferrous hydroxide (the results obtained in the laboratory of the presenting authors).

A TEM image of magnetite nanoparticles synthesized via the partial oxidation of ferrous hydroxide is shown in Figure 3. Specifically, the reagent solutions (ferrous sulfate hexahydrate, potassium nitrate, and potassium hydroxide) were mixed quickly and incubated at a temperature of 90 °C for 2 h in a nitrogen atmosphere to avoid the oxidation of the synthesized MNP. After the reaction was completed, the obtained MNPs were washed with water and separated magnetically.

A reaction in a constrained environment could be referred to as both a chemical and biological route of MNP synthesis with uniform dimensions, as various biological and synthetic reactors (templates) are used. They are protein cages of the iron storage proteins, ferritins, which are unique biomineralization systems [58]; phospholipid bilayers [59]; nanoporous materials with pore diameters from 2 to 50 nm [60]; stable dispersions of water/oil/surfactants [61], etc. Here we want to emphasize that, from our point of view, an ideal template for MNP synthesis is ferritin protein cages, as the protein protects newly formed MNPs from air oxygen oxidation and aggregation [62]. Moreover, a protein cage makes the newly synthesized MNP biocompatible, non-toxic, and biologically inert, so this synthesis method is usually utilized for MNP synthesis for biomedical applications.

Hydrothermal or high-temperature reactions are performed in autoclaves with high pressure and temperatures and allow one to obtain highly crystalline MNPs with various morphologies (sphere, cube, octahedron, etc.). The shapes/morphologies of synthesized MNPs are determined by reaction features, such as the reaction temperature, solvent type, precursor salt, reducing agent, etc. As the reactions are performed at pressures above 6000 Pa and temperatures above 200 °C [63], it means that researchers need expensive facilities for such syntheses, which is one of the disadvantages of this method.

MNPs with controlled shapes and sizes could be obtained via the polyol method. The shape, size, and yield of an MNP depend on the polyol type, salt ratio, salt concentration, etc. [63]. Polyols act as both reducing and stabilizing agents, which control particle growth and prevent the aggregation of particles. Despite the low cost of polyol synthesis, this method is thermally unstable and flammable.

The sol–gel method consists of two stages; the first is the hydrolysis and polycondensation of iron precursors with the formation of colloidal solutions of nanoparticles, and the second stage is the removal of the solvent (drying) with the formation of MNPs [64]. Among the advantages of this method are its environmental friendliness, the high purity and good crystallinity of the obtained MNPs, and the possibility of obtaining MNPs of various sizes simply by changing the annealing temperatures, while the average size of synthesized MNPs depends on the temperature in direct ratio. Among the disadvantages of this method are the usage of toxic organic solvents, the lengthy synthesis time (due to the drying process), and the possible contamination of the final product with reaction components.

Sonochemical synthesis (in which a chemical reaction occurs through the influence of ultrasonic irradiation) [65] accelerates the reaction due to the release of a huge quantity of energy in the process of acoustic cavitations in an aqueous reaction solution. The released energy creates high temperatures and pressures which increase the reaction rate and reduce the MNP growth time. The obtained MNPs are characterized by high crystallinity, saturation magnetization, and narrow size distribution. However, the shapes and sizes of the obtained nanoparticles are difficult to control, and the mechanism of this reaction is still not well understood.

One of the methods used for the biological synthesis of MNPs is bacteria-mediated reactions, which involve the production of MNPs via biologically controlled mineralization [66]. Biologically controlled mineralization leads to the formation of MNPs with uniform shapes and crystal sizes depending on the bacterial strains. The characteristics of biologically synthesized MNPs differ from chemically obtained nanoparticles in terms of particle size and morphology. Nonetheless, the yield of the obtained MNPs is low, and the synthetic mechanism needs further investigation.

Plant-mediated reactions consist of mixing iron precursors with a green substrate, which acts as both a reducing and stabilizing agent [67]. Various modifications of the concentrations of precursors and green substrate, time, temperature, etc., lead to MNPs with different characteristics. This process is environmentally friendly and simple and leads to the production of MNPs suitable for biomedical applications due to the non-toxic and biocompatible coating. However, the sizes and properties of the obtained MNPs are hard to control, and the particles produced are less stable and less homogenous.

To summarize, both top-down and bottom-up routes have advantages, disadvantages, and limitations, so the choice of synthesis method needs to be made in accordance with future applications of synthesized MNPs. From our point of view, biomimetic methodologies (inside a ferritin cage) or bacteria-mediated reactions are the most promising methods for MNP synthesis for the development of biosensors and POC diagnostics devices.

### 2.2. MNP Surface Coating

**Figure 4:**
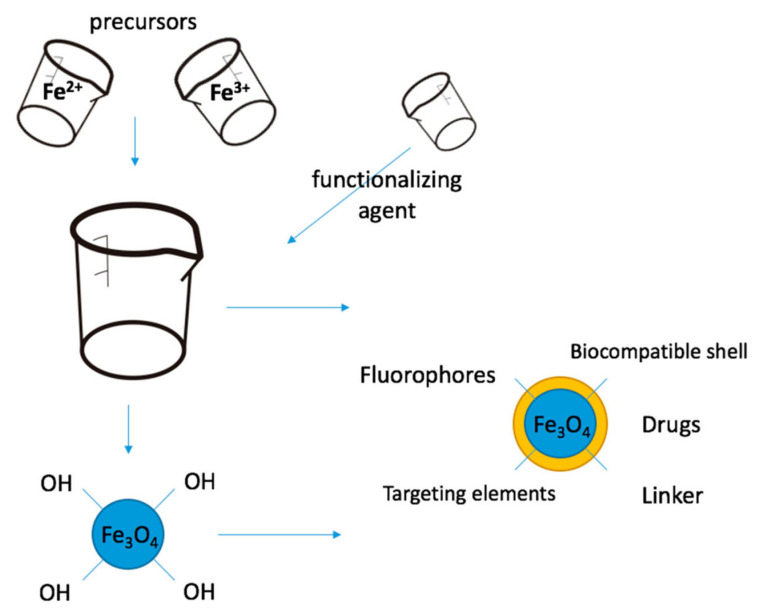
Schematic representation of the two main types of magnetite nanoparticle functionalization processes for medical applications: in situ and postsynthesis functionalization (reproduced without modification with permission from [69]).

To avoid the oxidation of freshly synthesized MNPs, to prevent their aggregation and agglomeration, and to make them biocompatible, their surface usually is coated with proper coverage (inorganic or organic) in situ or directly postsynthesis [68,69] (Figure 4).

Here, we briefly consider the main opportunities of such surface coating, which would allow us to tune the properties of MNPs.

#### 2.2.1. Silica

**Figure 5:**
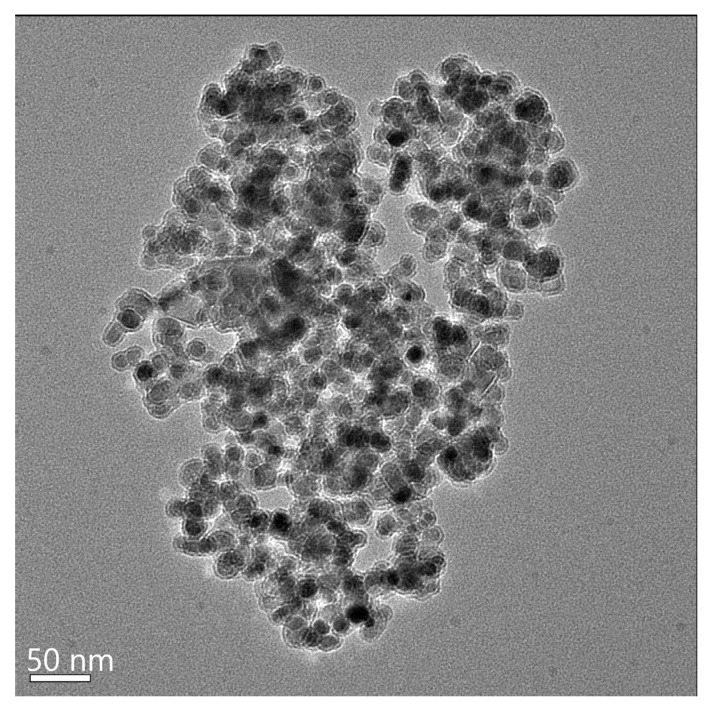
TEM-image of MNPs with silica coverage (the results obtained in the laboratory of the presenting authors).

Silica, as a coverage agent, has various functions, e.g., as a protecting agent, bio-compatible agent, adsorbent, drug carrier, etc. Due to its chemical stability and biocompatibility, the surface of silica can be easily functionalized with various biomolecules via the bio-conjugation process to obtain novel nanostructures for biomedical applications. Among the other methods of silica coverage (microemulsion [70], the formation of magnetite nanoparticles in the pores of mesoporous silica [71], etc.), the modified Stöber method [1,72,73] is the most widespread wet-chemical approach, in which the hydrolysis and condensation of tetraethylorthosilicate (TEOS) is utilized. In this method, freshly obtained magnetite nanoparticles are redispersed in a water–ethanol–ammonia mixture, followed by the addition of TEOS with continuous stirring/sonication and incubation. A TEM image of magnetite nanoparticles with silica coverage is shown in Figure 5. Magnetite nanoparticles were synthesized via the co-precipitation of ferric and ferrous chlorides, as described previously (Section 2.1.2). After washing the nanoparticles obtained with water and magnetically separating them, the nanoparticles were covered with silica via TEOS hydrolyzation in an alcohol–ammonia mixture. Various factors, such as the reaction temperature, incubation time, concentrations of TEOS and ammonia, pH, stirring method, etc., could affect the result. Among the advantages of silica coverage is the improvement of the long-term stability of the obtained nanoparticles and biocompatibility for further functionalization.

#### 2.2.2. Synthetic Polymers

Polymers are utilized in various biomedical applications because of their biomimetic properties and multi-functionality and are widely used for the coverage of MNPs [74,75]. Most polymers have various reactive groups in their structures, such as hydroxyl-, carboxyl-, and amino-groups, which could be used for the further bioconjugation of macromolecules [76]. Polymers could be chemically (covalent bonds) or physically (Van der Waals interaction) bound to the surface of synthesized MNPs. Among others, it is worth mentioning polyethylene glycol (PEG), which is a promising FDA-approved synthetic polymer for various biomedical applications [77]. The coverage of MNPs with polymers prevents their further oxidation and makes them stable, non-toxic, and biocompatible.

#### 2.2.3. Polysaccharides (Natural Polymeric Carbohydrates)

Polysaccharides are also types of polymer, and here we want to highlight natural biopolymers, such as chitosan, dextran, and starch, as biodegradable and biocompatible agents which have very low toxicity and have special properties. They are very abundant in nature, low cost, and easily modifiable [78]. Chitosan is derived from chitin, which is the main structure component of mollusks, insects, and fungi, and is widely used for the coverage of MNPs [79,80]. Chitosan is a natural polysaccharide that plays an important role in cross-linking with target molecules due to both the hydroxyl and amine groups in its structure. Dextran was originally derived from wine by Louis Pasteur. It is a complex branched polysaccharide and is extensively used to coat MNPs to make biocompatible and low-toxicity particles [78,81] for their further utilization as contrast agents for MRI and other clinical developments [82]. Starch is an abundantly biodegradable and inexpensive polysaccharide, which serves as an energy source in green plants and occurs naturally as insoluble semicrystalline granules; it consists of amylose and amylopectin. Among the advantages of MNP functionalization with starch are its low toxicity, abundance, and biocompatibility [78,83]. The special properties of polysaccharide-modified MNPs present opportunities to utilize them in various pharmaceutical and biomedical applications, such as in the treatment of hyperthermia, as an MRI contrast agent, for drug delivery, gene delivery, etc.

### 2.3. MNP Surface Functionalization

**Figure 6:**
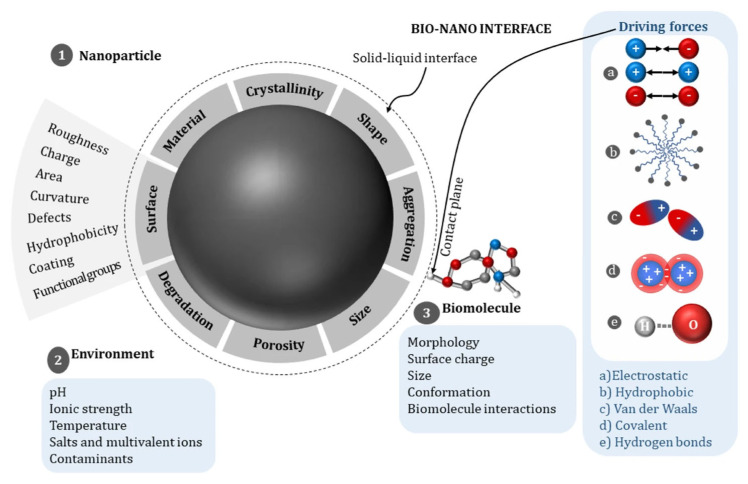
Frontiers constructing the bionano interface, which is constituted by the nanoparticle surface, the biomolecule, and the medium (reproduced without modification with permission from [44]).

Although MNPs with various surface coatings could be used for biomedical applications themselves, their further physical or chemical conjugation with macromolecules (peptides, nuclear acids, etc.) (Figure 6) provide various functional groups on the MNP surfaces, which change the chemical–physical characteristics of synthesized MNPs and endows them with biological properties [44], which could be utilized for the development of biosensors.

In the case of MNP functionalization with macromolecules, the surface chemistry of MNPs is also important. So, here, we briefly consider the main opportunities of such functionalization.

#### 2.3.1. Proteins, Peptides, and Enzymes

Generally, proteins, enzymes, and peptides are all biomolecules constructed by amino acids; although proteins are globularly or linearly functional of large structural biomolecules, enzymes are globularly functional proteins acting as catalysts, while peptides are small amino acid sequences. Amino acid successions predict the sizes, shapes, and functions of proteins and peptides. The main pathways involved in the interactions of proteins with MNP surfaces are electrostatic attraction/repulsion, hydrophobic attraction/repulsion, Van der Waals interactions, and coordinative bonding [84], and it has been shown that proteins bind with MNPs via carboxyl groups [85]. Despite the fact that the overall mechanism and driving forces of such interactions remain unclear [86], this has not hindered scientists from using proteins for MNP functionalization for the creation of new facilities for biomedical applications [87,88,89].

#### 2.3.2. Nucleic Acids

Nucleic acids are polymeric biomolecules constructed by nucleotides, and their main function is to store genetic information inside a cell. There are two major types of nucleic acid: single-stranded Ribonucleic acid (RNA) and double-stranded Deoxyribonucleic acid (DNA). Binding nucleic acids with MNPs follows the same mechanisms as binding with proteins: electrostatic interaction, hydrophobic interaction, and hydrogen bonding [90,91]. Many methods for MNP functionalization with nucleic acid have been developed [92] in order to produce various devices and systems for biosensing and POC diagnostics [93].

#### 2.3.3. Lipids

Lipids are a class of molecules with a wide variety of structures, but they have one common feature: they consist of hydrophilic headgroups and hydrophobic chains [94]. Their amphiphilic nature allows lipids to form a bilayer in aqueous environments, forming various structures, such as liposomes, micelles, and biological membranes. The formation of lipid coatings on MNPs’ surface is a dynamic process, driven by the minimization of MNPs’ high surface free energy and depending on MNP surface properties [95] but to understand the formation of the coating of MNPs with lipids, further investigations are required. The functionalization of MNPs with lipids facilitates the interaction of MNPs with the biological membrane and their passage through it, thus improving MNP compatibility for biomedical applications [96].

To conclude, surface coating and functionalization play important roles in the application of MNPs in biosensing because the surface coating of MNPs protects them from oxidation, avoids their aggregation, and improves their stability; in addition, further surface functionalization changes the physical-chemical properties of MNPs, increases their biocompatibility, and imparts biological properties to them. Mechanical and chemical stability is a big problem in biosensor development, so the proper surface coating and functionalization of MNPs lead to improving these parameters providing them with unique mechanical and chemical stability.

## 3. Diagnostic Application of MNPs

Due to the high selectivity and sensitivity of functionalized MNPs against target molecules and their unique magnetic characteristics, they could be utilized in a variety of diagnostic applications [12], i.e., sample preparation, biosensing, and POC diagnostic facilities; therefore, here, we focus on recent advances in this field of science and technology.

### 3.1. MNPs for Sample Preparation

The utilization of nanosized MNPs in sample preparation is based on their specific magnetic characteristics, namely, remote control and manipulation with an external magnetic field. This technique is rather facile and fast, while routine procedures of sample preparation are complicated, time consuming, labor-intensive (requiring sample centrifugation, filtration, etc.), and sometimes hazardous (requiring the use of toxic substances, etc.). The procedure consists of capturing target molecules (DNA/RNA, drugs, peptides) via target-specific magnetic particles and their further separation by applying an external magnetic field. This area of MNP application started to develop rapidly at the beginning of the 2000s, and today, commercial kits for macromolecule and organelle isolation and purification, etc. [97,98] are available and used in clinical practice.

**Figure 7:**
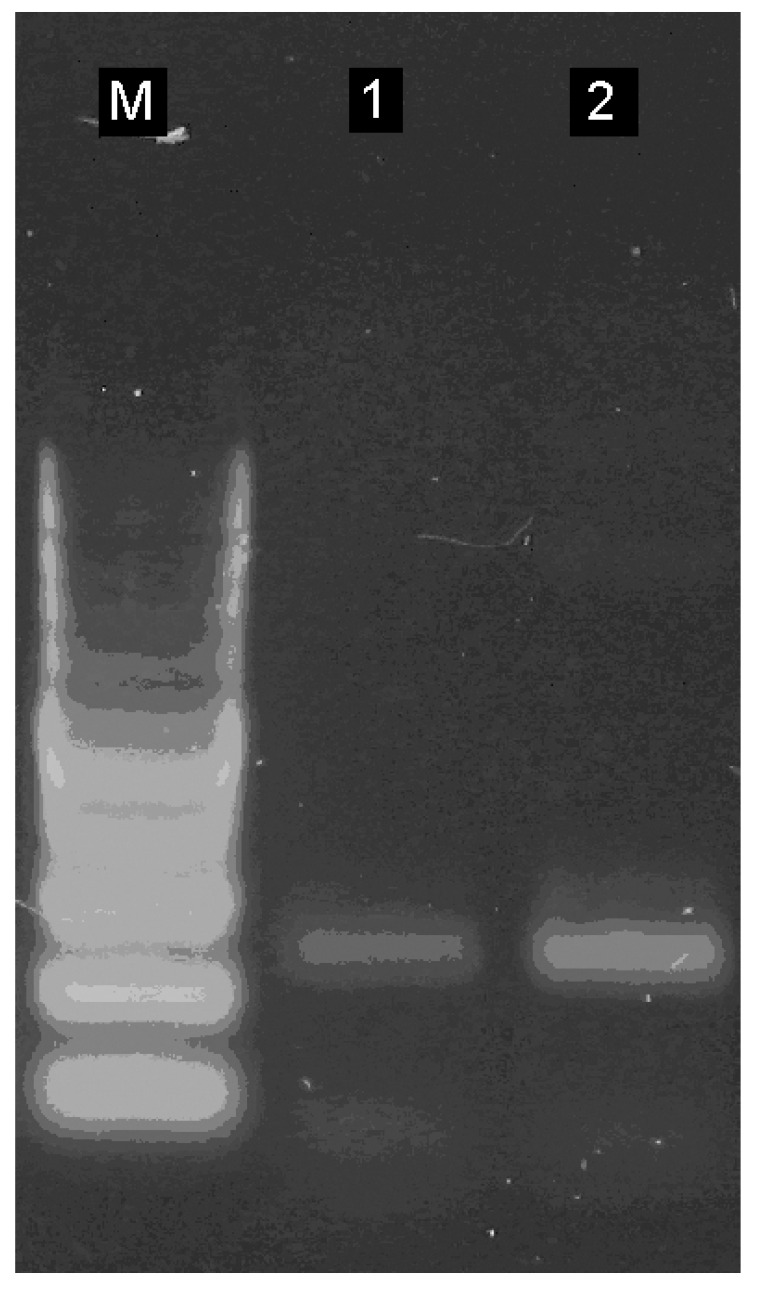
Comparison of the usage of commercial adsorbent (line 1) and synthesized silica-magnetite nanocomposite (line 2) for isolation of virus DNA fragments from sugar beet using agarose gel electrophoresis. Line M–DNA markers [100].

Dudchenko et al. synthesized silica-covered magnetite nanoparticles and used them for DNA isolation from different cell lysates [99,100]. It was shown that synthesized silica–magnetite nanocomposites were highly effective in the sorption of viral and bacterial native DNA/RNA from different biological objects for further amplification. The binding capacity of the synthesized silica–magnetite nanocomposites was much higher compared with a commercial adsorbent (Figure 7). The high purity of the obtained DNA/RNA was confirmed with the results of PCR amplification.

Sarkar et al. used carboxyl-coated magnetite nanoparticles for the isolation of RNA from breast cancer cells [101]. The authors mentioned that this method had the following advantages: it is rather simple, inexpensive, and does not require complicated manipulation of the samples, such as extraction and centrifugation. DNA isolated using this process can easily be used in further experiments. Fan et al. used a modified solvothermal method for the synthesis of highly magnetic silica–magnetite nanoparticles for DNA purification and showed high extraction and elution yields [102]. The authors expected that the synthesized silica–magnetite nanocomposites could be used as novel separation, immobilization, and absorption materials. It should be mentioned that the proper surface functionalization of MNPs is crucial for obtaining reliable results [103].

Functionalized MNPs could be used for drug detection in various biological fluids. Yang et al. developed a TiO_2_-modified mesoporous magnetite nanoparticle for the detection of commonly used drugs in human blood [104] using a liquid–liquid extraction procedure. The authors concluded that the proposed procedure using modified magnetic nanoparticles has some advantages, among them being the minimization of blood matrix effects during analysis. Moreover, the synthesized silica–magnetite nanocomposites have a high adsorption capacity, are not expensive, and can be easily manipulated. Jalilian et al. used mesoporous magnetite nanoparticles for the simultaneous extraction and quantification of drugs with different polarities [105]. They used a combination of the magnetic solid phase extraction method and dispersive liquid–liquid microextraction (MSPE–DLLME) and concluded that the advantages of these extraction methods, such as their simplicity and rapidity, are acquired due to the usage of created nanocomposites which can be easily collected from the solution via an external magnetic field. Bozyigit et al. [106] used polystyrene-coated magnetite nanoparticles as adsorbents of antidepressant drug ingredients with high yields, with their further determination via gas chromatography-mass spectrometry.

Nowadays, the applications of NMPs for sample preparation are being rapidly developed, and this trend is moving towards automation, efficiency, and POC detection [107].

### 3.2. MNPs in Biosensing

**Figure 8:**
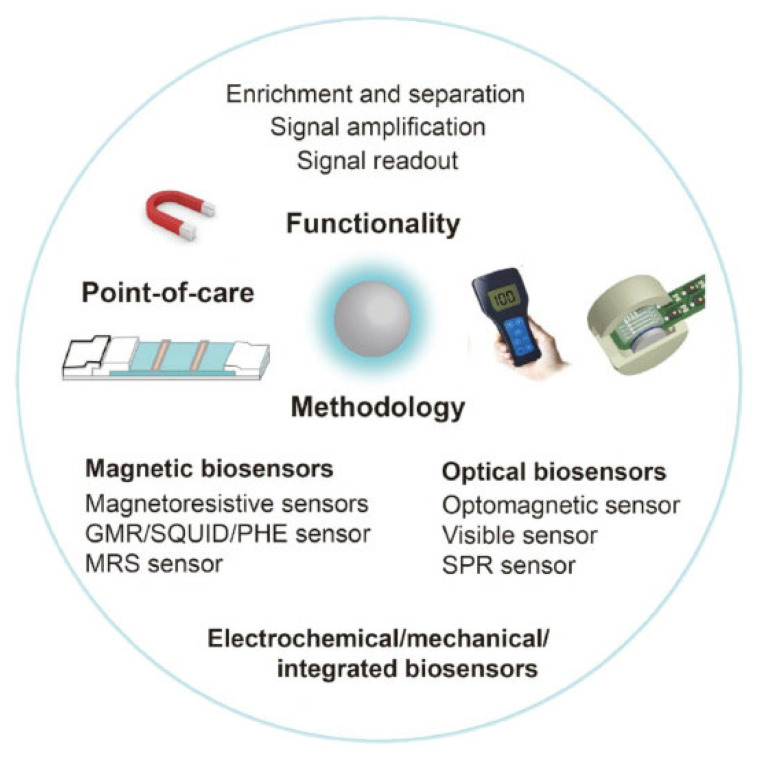
An overview of the functionality and methodology of MP-based biosensors for point-of-care testing (reprinted with permission from [111]).

The first biosensor was invented in 1956 by Leland C. Clark for oxygen detection (today, it is well known as the Clark electrode) [108]. Ever since, the field of biosensors has developed strongly and continues to progress rapidly. Recently, biosensors have attracted considerable interest in various areas of science and applications. The main characteristics of biosensors are their high selectivity, which determines by the ability of a bioreceptor to detect a specific analyte in a sample with admixtures and contaminants; reproducibility (the ability of the biosensor to generate identical responses for multiple experiments); stability during ambient disturbances; and sensitivity [109]. The main principle of biosensor functioning is to recognize a chemical/biological reaction using a bioreceptor and to convert it to a quantifiable signal, which is proportional to the analyte concentration in the reaction. The conversion of a biorecognition event into a quantifiable signal is achieved by means of a transducer, which utilizes the following sensing technologies: electrochemical, optical, mechanical, magnetic, mass-sensitive, thermal, etc. Among the MNP-derived biosensors, we can highlight devices based on electrochemical, optical, and magnetic sensing technologies as the most widely used in combination with magnetite nanoparticles [110] (Figure 8).

Electrochemical sensing technology exploits changes in reduction/oxidization reactions and electron transfer properties associated with the binding of a target biological object to a bioreceptor [112]. In this analytical platform, properly functionalized MNPs are immobilized on the surfaces of various electrodes, enhancing their loading capacity and electron/reactants transfer due to MNPs’ special properties, such as mechanical stability and proper functionalization, which leads to an essential increase in the sensitivity and selectivity of the electrode. Afzali et al. [113] developed and fabricated a novel electrochemical sensor based on a glassy carbon electrode decorated by a molecularly imprinted polymer-coated magnetite–graphene oxide nanocomposite (Fe_3_O_4_@GO), which was successfully applied to the selective determination of the anticancer drug, capecitabine, in human plasma, and pharmaceutical samples. It was shown via TEM that magnetite NPs have an average diameter of about 280 nm, and the layer of a graphene oxide shell-covered magnetite core is about 5 nm thick. The authors claimed that the developed sensor has high selectivity, sensitivity, reproducibility, and stability. Tian et al. [114] developed an ultrasensitive electrochemical magnetite-based biosensor for RNA detection. The magnetite in the created biosensor was involved not only in the separation and enrichment of the target molecules under a magnetic field but also in the improvement of the sensitivity of the biosensor. The authors concluded that the proposed method is easy and highly reproducible, which makes it promising for utilization in biosensor-based research and diagnostics. Sun et al. [115] proposed a modified electrode based on magnetite nanoparticles covered with a molecularly imprinted polymer for the electrochemical recognition of bovine hemoglobin (BHb). Magnetite nanoparticles covered with silica were functionalized with dopamine and BHb as a template molecule. The biosensor showed high sensitivity, good selectivity, and stability in the detection of BHb. Gold/N-trimethyl chitosan/magnetite (Au/TMC/Fe_3_O_4_) nanocomposites were employed for the development of ultrasensitive biosensors such as a urine albumin electrochemical biosensor [116], an epidermal growth factor receptor (EGFR) electrochemical biosensor [117] for early cancer diagnosis, a chip-based sandwich electrochemical genosensor for the quantitative assessment of RASSF1A tumor suppressor gene methylation [118], and an electrochemical genosensor for the ultrasensitive detection of microRNA [119].

**Figure 9:**
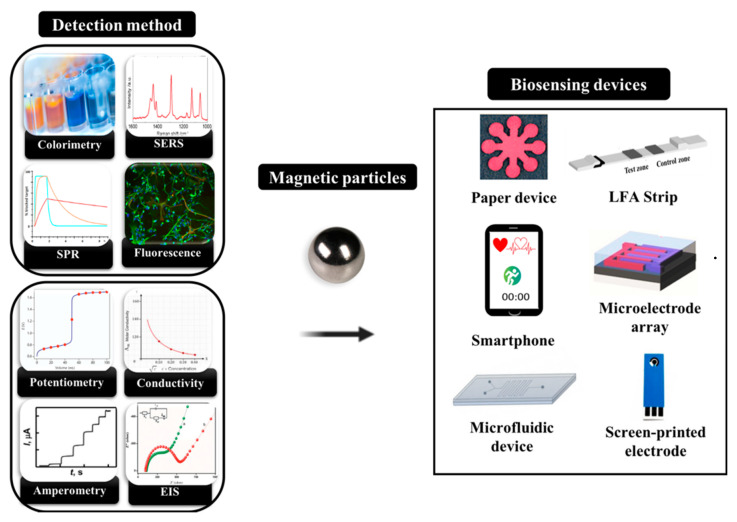
Schematic representation of the detection method based on optical and electrochemical techniques (Colorimetry, Surface-enhanced Raman spectroscopy (SERS), Surface Plasmon Resonance (SPR), Fluorescence, Potentiometry, Conductivity, Amperometry, and Electronic Impedance Spectroscopy (EIS)) combined with magnetic materials for biosensing (reprinted with modification with permission from [12]).

Optical sensing technology identifies changes in optical parameters (color/refractive index/fluorescence/absorbance/surface plasmon resonance/etc.) while the target analyte binds the bioreceptor [120]. In this analytical platform, properly covered and functionalized MNPs change their optical activity depending on the presence/absence of a target biological object, which is detected via fluorescent emission/quenching or other optical changes. The increased sensitivity and selectivity of the analytical platform is achieved through the high specific surface of the nanoparticles and the synergistic optic effects of magnetite and proper surface coverage (Au, CDs, etc.). Zarei-Ghobadi et al. [121] reported a novel and cost-effective Au-capped magnetite-based sensor for the detection of short DNA/RNA fragments of Human T-lymphotropic virus type 1 (HTLV-1) for the first time. The main operating principle of the created sensor lay in the quenching of carbon dot fluorescence, adsorbed on the surface of Au-capped magnetite in the absence of a target, while in the presence of a target, the fluorescence was recovered. Zhang et al. [122] described fluorescent magnetite-based carbon dots conjugated (spore@Fe_3_O_4_@CDs) nanocomposites as highly efficient biosensing devices for the direct real-time detection of toxins secreted by *Clostridium difficile*, a bacterium that causes infection in the large intestine. It was shown that the synthesis of nanocomposites is rapid, simple, mass-produced, and cost-effective and consists of the step-by-step encapsulation and functionalization of porous natural spores. Xiong et al. [123] reported a microfluidic device based on magnetic nanochains, which was built of superparamagnetic Fe_3_O_4_ nanoparticles with 250-nm diameters that used surface-enhanced Raman scattering for signal transduction. They demonstrated the ability of the created device to rapidly (8 min), sensitively, and accurately detect cancer biomarkers in serum samples as well as bacteria in human saliva in a volume ~1 μL of body fluids, in contrast to the routine, time consuming procedures. A schematic representation of the magnetic materials-based biosensing devices that use optical and electrochemical detection methods is shown in Figure 9.

**Figure 10:**
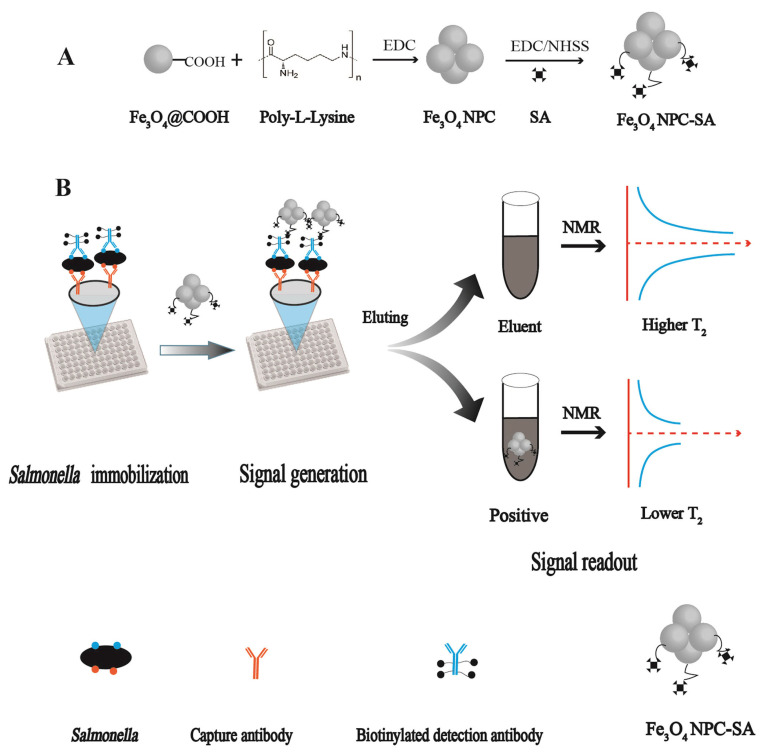
NMR biosensor based on nuclear magnetic resonance for detection of Salmonella: (**A**) representation for preparation of Fe_3_O_4_ nanoparticles (NP) and Fe_3_O_4_-streptavidin nanoparticles (NPC-SA); (**B**) overview of NMR biosensor for detection of Salmonella in milk (reprinted with permission from [124]).

Magnetic sensing technology detects changes in the magnetic properties (Giant Magnetoresistance/nuclear magnetic resonance imaging (NMI)/etc.) of target analytes related to their binding to a biosensor. In this analytical platform, the magnetic properties of MNPs are utilized and detected directly, as changes in the analyte concentration lead to changes in the magnetic properties of the developed nanoparticles. Today, MNPs with various types of surface functionalization are widely studied as positive (T_1_) contrast agents as an alternative to the gadolinium contrast agent for NMI contrast enhancement. Zou et al. [124] developed a magnetite nanoparticle-based biosensor, which utilizes the technique of nuclear magnetic resonance for Salmonella detection. To improve the sensitivity of the created NMR biosensor, the authors adopted two signal amplification methods (a streptavidin-biotin system and a magnetic nanoparticle cluster), which led to a significant increase in the sensitivity of the created biosensor (Figure 10). The conclusion was made that the created biosensor could serve as a prospective instrument for the sensitive and fast detection of pathogens in samples of various origins. Zhao et al. [125] developed a method for the accurate and reliable detection of *Listeria monocytogenes* in food based on functionalized iron/magnetite nanoconjugates, which change their properties depending on the binding of the antibody on the surface of the nanoparticles. It was shown that the specific binding of *L. monocytogenes* and antibody-modified nanoparticles leads to the aggregation of biofunctionalized MNPs, which entails the change in the relaxation time of surrounding nanoparticle water protons that could be detected by nuclear magnetic resonance. The high specificity, sensitivity, and rapidness of the method were shown in the detection of *L. monocytogenes* using NMR. Mu et al. [126] effectively combined a tunneling magnetoresistance (TMR) biosensor with a magnetic immunochromatographic test strip for magnetic signal detection. They developed a new method for ricin (a highly potent toxin) detection via the identification of the magnetic field intensity of a magnetic signal probe on an immunochromatographic test strip and realized the rapid quantitative detection of toxin containing samples. The advantages of the developed method, compared with traditional routine methods, based on optical signals, are high sensitivity and specificity, good reproducibility, simple operation, and accurate quantification.

Wu et al. [127] described a new method for the quantitative detection of molecular biomarkers, which utilizes the Brownian relaxation of magnetite-based nanoparticles. In magnetic particle spectroscopy (MPS) measurements, the authors used the harmonics of oscillating MNPs, which could be collected from microquantities of magnetite nanoparticles within 10 s and correspond to the bound states of the MNPs. As the model system, the authors used streptavidin/biotin molecules with ultrahigh binding affinity and demonstrated the validity of the created method for rapid, point-of-care, sensitive, and versatile immunoassays. The results show that by using the developed method, one could detect biological objects directly from investigated biological samples, and the procedure requires minimum sample preparation.

We could conclude that using biomolecules in combination with MNPs provides great advantages in biosensor development due to synergistic effects and the result of the more sensitive, rapid, and accurate identification and quantification of target biological objects.

### 3.3. MNPs for POC Diagnostics

MNPs with proper surface functionalization are especially suitable for the development of lab-in-chip biosensors for easy, reliable, and cost-effective POC diagnostic facilities. In addition, MNPs are widely used in the molecular diagnosis of human viruses [128] and other infectious pathogens [129]; MNP-based biosensors are also an extremely promising form in highly accurate POC SARS-CoV-2 detection [130,131,132]. In this review, we briefly highlight the following biological objects, which have been recently analyzed using MNP-based POC devices: SARS-CoV-2, Human chorionic gonadotropin (hCG), the typical oral pathogen of Streptococcus, foodborne bacteria in food and environmental samples (*Listeria monocytogenes*, *Campylobacter jejuni*, and *Staphylococcus aureus*), pathogenic bacteria (*Escherichia coli* (*E. coli*), and *Staphylococcus aureus* (*S. aureus*), the food pathogen *Vibrio parahaemolyticus*), the bacteria *Acinetobacter baumannii*, and influenza A H1N1 virus (Table 1).

The problems in the analysis of various viruses and pathogens via alternative methods are as follows: the methods are time consuming, usually involving the complex pretreatment of samples (purification, centrifugation, etc.), which leads to the need for complex equipment, and as a result, the price of the analysis is high. In some cases, the analysis could be incorrect due to the matrix effect of some admixtures. Moreover, specialized, well-trained staff are required to perform such analyses.

Utilization of properly functionalized MNPs for POC diagnostics of target biomolecules (glucose, lactate, etc.) is mostly based on enzymatic-mediated reaction with hydrogen peroxide formation. The combination of various peroxidases, MNPs, and bacteria/viruses could be used for the catalysis of glucose into hydrogen peroxide with its further determination and, respectively, biological object concentration determination. MNPs, on their own, could mimic peroxidase activity via the Fenton reaction due to the presence of Fe^+2^ and could be attributed to the class of nanomaterial-based artificial enzymes (nanozymes), that are very promising for state-of-the-art POC diagnostics. Such peroxidase mimicking activity retained after macromolecule bioconjugation could be one more merit.

**Figure 11:**
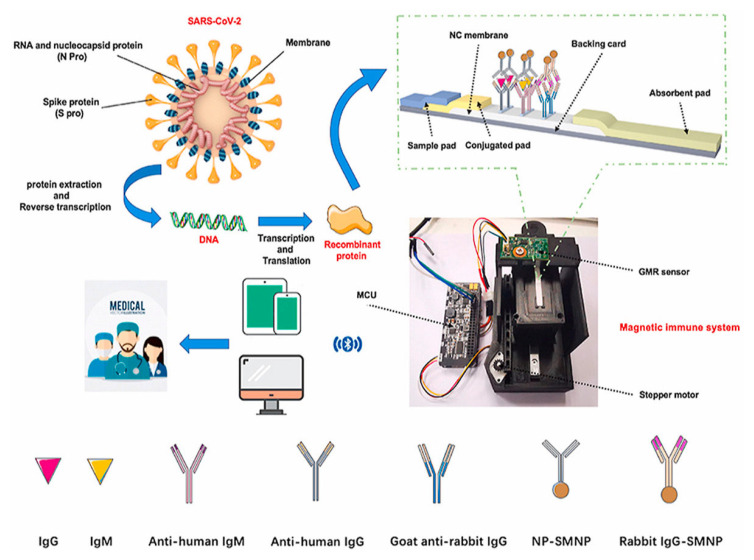
Composition of SARS-CoV-2 virus, the scheme for detection of anti-SARS-CoV-2 immunoglobulin M (IgM) and G (IgG)using the test strip, the magnetic immune system device, and medical applications (reprinted with permission from [134]).

Here, we would like to highlight several magnetite-based biosensors for the detection of SARS-CoV-2 for POC diagnostics [133]. Bayin et al. [134] developed a lateral flow immunoassay (LFIA) method for the simultaneous quantitative detection of anti-SARS-CoV-2 immunoglobulin M (IgM) and G (IgG), which is based on magnetite and a giant magnetoresistance (GMR) sensing system. The average MNP core size was around 70 nm, and the saturation magnetization was about 62 emu/g, which means that synthesized MNPs had strong magnetic responses. Authors anticipated that their LFIA test strip could be used by clinicians to obtain preliminary test results, correctly analyze infections, and create a proper treatment strategy (Figure 11). In comparison with other detection methods, this method is cheaper, faster, and more convenient.

**Figure 12:**
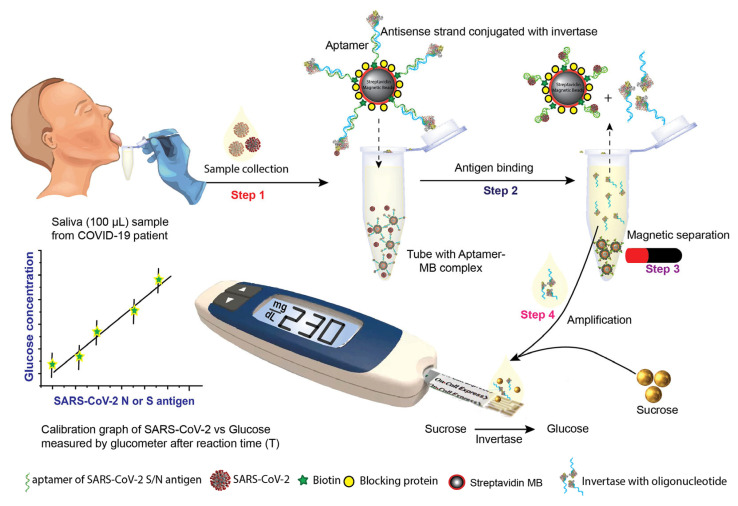
Overview of proposed POC, SARS-CoV-2 N, or S protein-specific biotinylated aptamer-based COVID-19 assay. In the first step, saliva is added to this cocktail. In the second step, the viral antigen or the SARS-CoV-2 virion binding is released. In the third step, the magnetic separation of magnetic nanoparticles conjugated to the aptamer-antigen complex is used. In the final step, the remaining solution is collected and incubated with sucrose. Invertase, which is contained in the solution, converts sucrose to glucose which can be directly measured using a portable glucometer. The glucose concentration is correlated with the SARS-CoV-2 N or S protein concentration (reprinted with permission from [136]).

For the first time, Li et al. [135] demonstrated a rapid (55 min) and highly sensitive method for the determination of the SARS-CoV-2 N protein in whole serum. They used a dually labeled, carboxylated magnetite nanoparticle-based immunosensor. This developed immunosensor does not require external pumps or power sources and can be adapted for use on a smartphone-based diagnostic device. The authors concluded that the developed immunosensor demonstrated portability, simplicity, and high sensitivity, which means it has the potential for use in POC COVID-19 testing. Singh et al. [136] reported a POC system composed of a glucometer, which uses streptavidin-coated magnetite-based nanocomposites and an aptamer-based SARS-CoV-2 salivary antigen assay (Figure 12). The developed system only employs low-cost reagents, and the cost of one test is equal to USD 3.20. Moreover, the developed test is rapid, highly sensitive, and can be used to identify SARS-CoV-2 infection in patients’ saliva within 1 h. So, the proposed approach provides a low-cost, fast, and accurate diagnostic tool for the distributed assessment of SARS-CoV-2 infection at scale.

**Figure 13:**
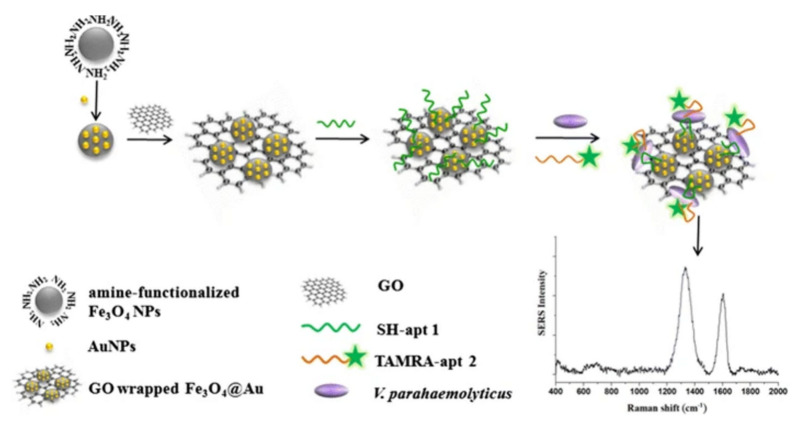
Schematic of SERS aptasensor based on graphene oxide (GO)-wrapped Fe_3_O_4_@Au nanostructures for *V. parahaemolyticus* determination (reprinted with permission from [141]).

Kim et al. [137] synthesized Pt-decorated magnetite nanocomposites with various Pt/magnetite ratios and integrated them into lateral flow immunoassay strips as magnetically separable probes. The efficiency of the created nanocomposites was tested via the detection of the model biomarker, Human chorionic gonadotropin (hCG), a biomarker of pregnancy. The Pt-decorated magnetite nanocomposites were proven to have high catalytic activity due to their high catalytic properties. The authors demonstrated that the created bioassay device has a sensitivity higher by two orders of magnitude than that of the traditional gold-based nanoparticle lateral flow immunoassay. These results indicate that synthesized Pt-decorated magnetite nanoparticles, combined with lateral flow immunoassay strips, enable effective POC detection with ultra-low detection limits in medical diagnostics. Zhang and co-authors [138] developed a Fe_3_O_4_-DNA-engineered nanozyme interface for the rapid and sensitive detection of dental bacteria and showed that magnetite nanoparticles greatly improve DNA-bacteria interactions. The authors concluded that the created DNA-magnetite nanoconjugates were acceptable due to their high affinity and the immediate detection of the typical oral pathogen of Streptococcus. This interface creates new possibilities for producing a POC device for rapid clinical caries screening in future and demonstrates wide ranging potential in the clinical diagnosis of dental diseases. Tu et al. [139] reported a universal surface-enhanced Raman scattering (SERS)-based lateral flow assay (LFA) using wheat germ agglutinin-modified, Au-modified magnetite nanocomposites for the detection of foodborne bacteria in food and environmental samples with high sensitivity. The proposed universal detection method was effectively utilized for the accurate and specific detection of three foodborne bacteria with a low detection limit (10 cells mL^−1^) and a short testing time (<8.14%). The authors concluded that this approach could be easily expanded for the detection of various pathogenic bacteria by only replacing the detection antibody on the test strip. Thus, this strategy can be transformed into a powerful instrument for the universal and on-site recognition of various pathogens in the future. Modified Fe_3_O_4_@Au nanoparticles were also utilized for the development of sensitive SERS platforms for the detection of pathogenic bacteria (*Escherichia coli* (*E. coli*), *Staphylococcus aureus* (*S. aureus*) and food pathogen *Vibrio parahaemolyticus*) [140,141] (Figure 13). It was shown that the created sensitive platforms have potential applications in the accurate diagnosis of pathogens.

Su and co-authors [142] developed an integrated electromagnetically driven microfluidic system for the rapid POC detection of *Acinetobacter baumannii* (AB). The developed device comprises magnetite-based nanocomposites with AB-specific aptamers that are used to capture bacteria, and secondly, aptamer-bound quantum dots (QDs) are incorporated into the device for the quantification of the number of bacteria with a light emitting diode (LED)-induced fluorescence unit. This system offers fast (up to 30 min), sensitive, and fully automated AB detection with low energy and chemical consumption (10 μL of sample and chemicals) by using a dual-aptamer assay. The authors concluded that the system would allow for the rapid clinical diagnosis of AB in the near future. Lu et al. [143] introduced a new type of digital microfluidic facility for H1N1 virus detection, which utilizes a one-aptamer/two-antibody assay on magnetite-based nanoparticles. Aptamers, which have high specificity towards H1N1 viruses, were conjugated with magnetite-based nanoparticles such that they could be specifically captured and detected. The reaction time of the complete analysis using the created facility was less than 40 min, and the volume of all the reagents, including the sample, antibodies, and wash buffers, was about 20 μL. The authors concluded that the presented digital microfluidic facility enables a complete diagnostic process to be carried out for influenza A H1N1 viruses, which could be promising for the fast and correct diagnosis of influenza.

The analytical merit of approaches to the detection of bacterial and viral infections using magnetite-based POC devices is their high sensitivity and specificity compared with conventional diagnostics methods. In addition, the proposed methods are relatively quick (they could be performed in minutes), while conventional methods require long operation times, i.e., a polymerase chain reaction requires hours (3–24 h), and serological assays require days (3–10 days). One more merit of the proposed POC devices is the small amount of sample required for the analysis (μL). To summarize, MNPs are multifunctional materials and promising platforms for the creation and development of biosensors and POC diagnostic devices, which provide fast, inexpensive, and accurate diagnostics of both bacterial and viral infections.

## 4. Perspectives of MNPs for Molecular Logic

Molecular logic gates, built on biomolecules, are becoming an alternative to logic operations based on silicon. A variety of molecular switches, known as “molecular logic gates”, respond to various input signals in accordance with the principles of Boolean logic. The most fundamental logic gates include single-input (“YES”, “NOT”, “PASS 0”, and “PASS 1” logic gates), double-input (“OR”, “AND”, “XOR”, “INHIBIT”, “NOR”, “NAND”, “XNOR”, and “IMPLICATION” logic gate), and multiple-input logic gates. In our previous works, we manufactured gold nanoparticles-based structures that can realize “AND”, “OR”, “NAND”, “NOR”, “XOR”, and “XNOR” logic gates and are responsive to two separate inputs: surrounding pH and proteinase availability [144]. We discussed the applicability of fluorescence as a means of logic gate implementation [145] and various logic functions based on carbon dot sensing, with a special emphasis on recent advancements in CD-based logic gates, as well as on the understanding of the mechanism of CD-based logic structures [146]. We designed nanocomposites based on gold nanoparticles with red emitting carbon dots which can be used for the creation of a fluorescence lifetime imaging microscopy (FLIM)-based logic gate which can act in response to multiple input parameters [147]. MNPs, which are susceptible to external magnetic fields, possibly have the widest range of uses in various fields, including logic applications, because of their special features. MNPs (magnetic beads), in combination with DNA, were first used by Leonard M. Aldeman in 1994 to carry out computations at the molecular level [148] (since then, DNA has become a reference in biomolecular computing). Currently, scientists utilize MNPs in combination with various substances for the creation of new molecular logic devices.

**Figure 14:**
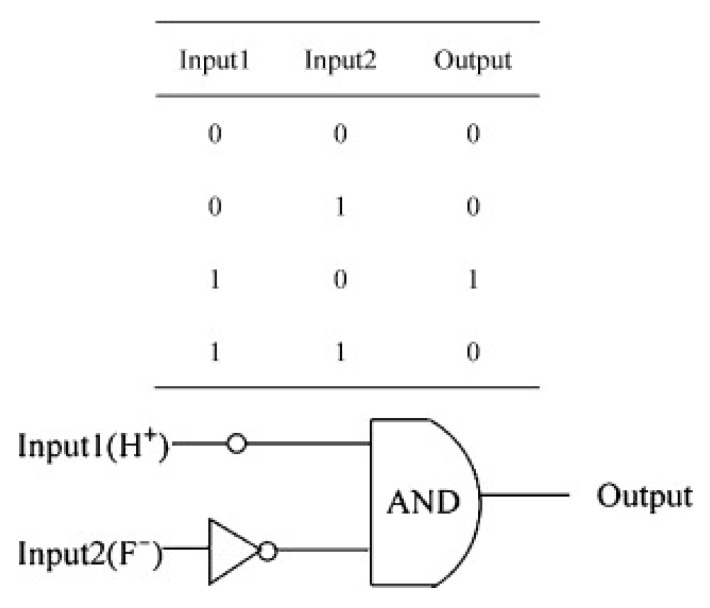
General description of an INH gate and the subsequent truth table (0 means low signal, 1 means high signal) (reprinted with permission from [149]).

Molecular logic element–modified multifunctionalized magnetite–silica nanoparticles have been studied by X. Tian et al. [149]. To produce the hybrid material NAU-Fe_3_O_4_@SiO_2_, 1,8-naphthalimide fluorophore was combined with silica–magnetite nanocomposites and amine and urea receptor units (NAUs). It was observed that the created nanocomposites exhibited relatively high saturation magnetization, coercivity, and remanence at 300 K, which indicated they have superparamagnetic behavior. At the molecular level, the fluorescence intensity signal was used as the output of an INHIBIT (INH) logic gate, which takes advantage of the fluorescent changes that a hybrid material undergoes when cations (H^+^, F^+^, and their mixture) are added (Figure 14). It is anticipated that the INH logic gate will function at the molecular level. A new generation of digital gadgets might be developed using this simple organic-inorganic hybrid material.

**Figure 15:**
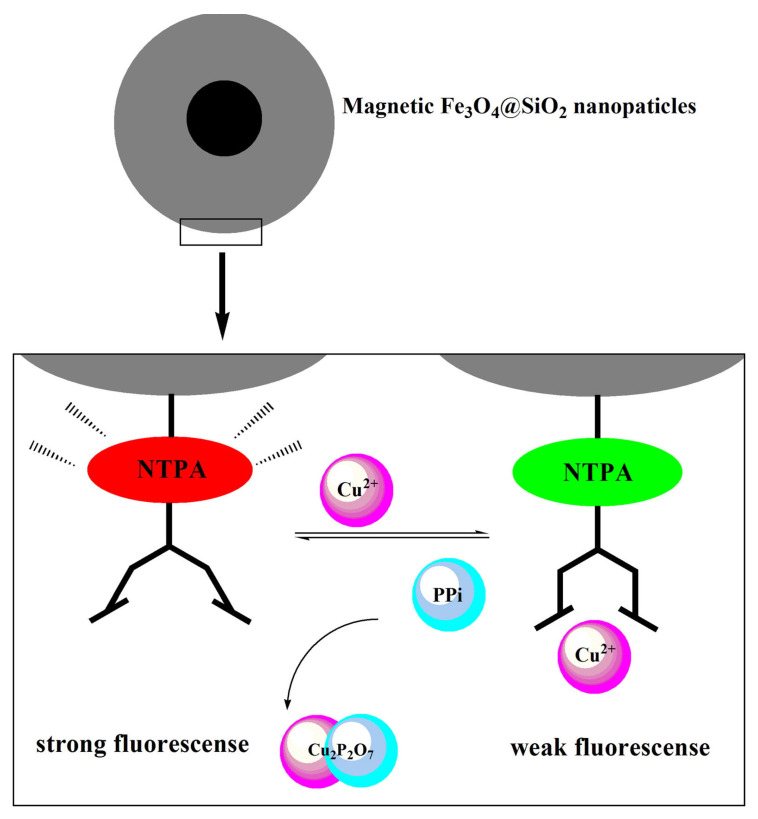
Proposed mechanism of fluorescent chromophore NTPA for assay of Cu^2+^ and PPi (reprinted with permission from [150]).

Similar to this, the same authors also built a molecular logic gate system (NTPA-Fe_3_O_4_@SiO_2_) that uses pyrophosphate (PPi) and Cu^2+^ as inputs and is based on multifunctional magnetic silica nanoparticles [150] (Figure 15). They showed that at the nanoscale level, the fluorescence emission variations in NTPA-Fe_3_O_4_@SiO_2_ caused by the inputs of Cu^2+^ and PPi can be seen as an IMPLICATION logic gate.

S. Zhang and his group created an easy and all-purpose platform based on the properties of gold–silica–magnetite–based magnetic beads (Fe_3_O_4_@SiO_2_@Au) and DNA for the construction of numerous logic gates [151]. The half adder and half subtractor represent DNA-conjugated gold nanoparticles immobilized on magnetic nanoparticles. The new feature of the created system is the ability of the inputs to interact with silica-magnetite-gold nanoconjugates. The ability to reset the logic operations via the thermal denaturation and magnetic separation of the computing modules is another innovative feature. Additionally, the created half adder and half subtractor share a constant and are implemented on a basic DNA/magnetic bead platform in an enzyme-free system. In the next study carried out by this group, the researcher developed a magnetic bead/DNA (Fe_3_O_4_@SiO_2_@Au) system to build a library of logic gates, which allow the detection of multiplex target miRNAs [152]. The miRNA-catalyzed hairpin assembly (CHA) was effectively used in this assay to design extremely sensitive multiplex detection systems by building two/three input concatenated logic circuits with great selectivity. The creation of quick and intelligent detection was greatly facilitated by the fact that a hairpin-based recognition structure can differentiate particular target miRNAs (such as miR-21, miR-155, and miR let-7a) under a logic function control. The recognition structure can be reset by the thermal denaturation of the output system and the magnetic separation of the computer modules. As a result of the work, the cascade INHIBIT-OR logic gate was modified on the surface of gold nanoparticles which were immobilized on magnetic beads, which utilized the quenching properties of gold nanoparticles toward fluorophores, as well as the magnetic properties of magnetite nanoparticles.

**Figure 16:**
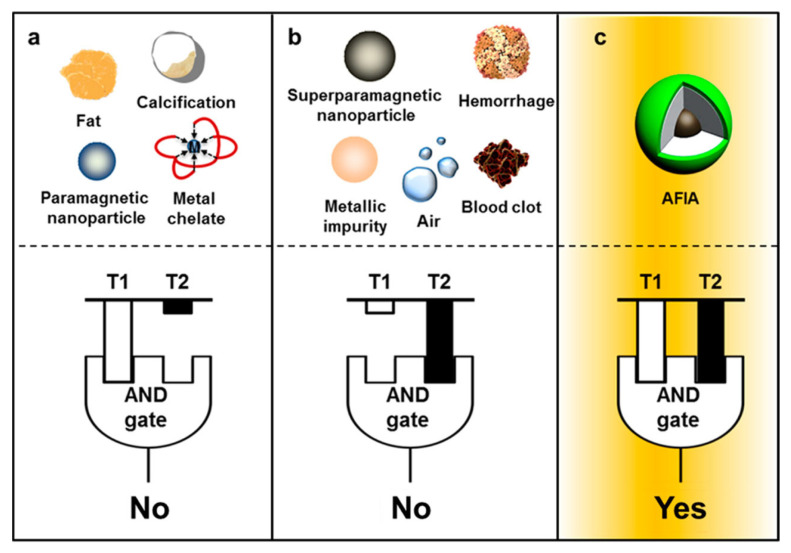
Illustration of artifacts and typical contrast agents that give high MRI contrast effects in either T_1_ or T_2_ but do not satisfy AND logic (**a**,**b**) and artifact filtering imaging agent (AFIA) that fulfills AND logic (**c**) (reprinted from [153] with permission of ©2014 American Chemical Society).

Cheon and colleagues created a method that effectively removes MRI abnormalities caused by blood clots, fat, air bubbles, and other conditions by employing magnetically decoupled T_1_-T_2_ dual-mode nanoparticle contrast agents [153]. This research team created a variety of T_1_-contrast-materials core-shell nanoparticles with a core made of T_2_ contrast magnetic nanoparticles (such as MnFe_2_O_4_ or Fe_3_O_4_) and separated the SiO_2_ layer. T_1_ and T_2_ imaging modes were used to create an AND logic gate (Figure 16).

**Figure 17:**
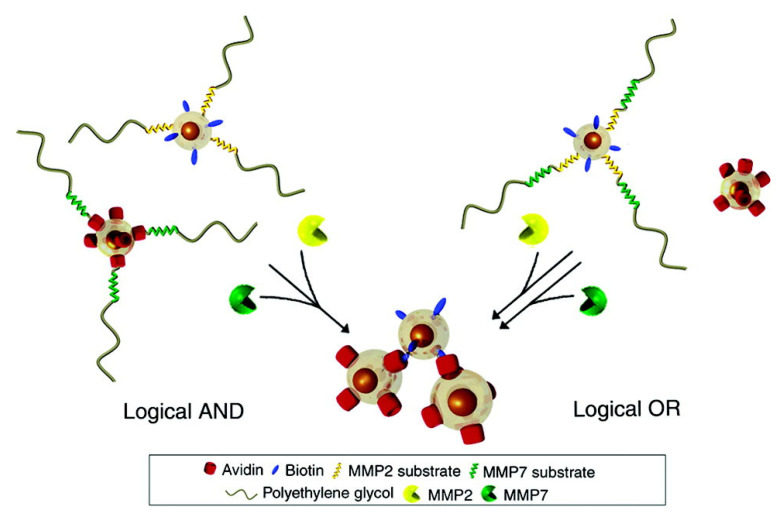
Schematic representation of the assembly of nanoparticles to construct logical AND (left) or logical OR (right) by attachment of protease removable polyethylene glycol polymers (reprinted from [154] with permission of ©2007 American Chemical Society).

Using a unique technique, the resulting T_1_ and T_2_ images with their associated T_1_ and T_2_ artifacts were postprocessed to create a composite T_1_/T_2_ image. Superparamagnetic Fe_3_O_4_ nanoparticles were self-assembled logic-gated by inputs related to cancer invasion, according to Bhatia et al. [154]. Avidin- and biotin-modified superparamagnetic Fe_3_O_4_ nanoparticles were synthesized. Following this, oligopeptide linkers were used to pegylate each type of nanoparticle by connecting their surfaces to PEG. The authors used MMP2 and MMP7 proteases as inputs and pegylated avidin-modified nanoparticles with PEG via an MMP7 protease substrate linkage and biotin-modified nanoparticles with PEG via an MMP2 protease substrate linkage to construct an AND logic gate. As a result of the peptide linker’s cleavage with this input, nanoparticles that were previously shielded by the PEG corona were no longer protected. The simultaneous detachments of PEG coronas from both varieties of nanoparticles only resulted in their assembly when both inputs were present, which amplified the proton’s T_2_ relaxation rate. Using avidin-modified nanoparticles without PEG corona and biotin-modified nanoparticles pegylated via both MMP2 and MMP7 substrate linkers, an OR logic gate was developed. In this instance, the presence of one or both inputs caused the PEG corona to separate, which was followed by the assembly of nanoparticles (Figure 17).

**Figure 18:**
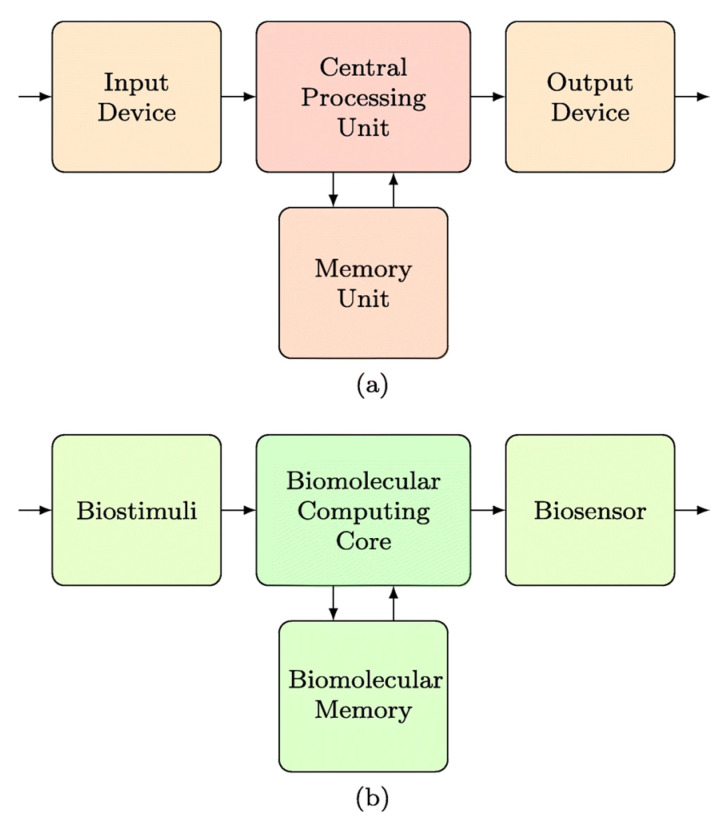
A standard architecture for a computer design (**a**) and a proposed biomolecular computing structure (**b**) (reprinted with permission from [155]).

Aiassa and co-authors [155] proposed an architecture for computer design (similar to Von Neumann architecture [156]) composed on a biomolecular computing system, which included biosensors as output devices and was based on molecular logic gates (Figure 18); both designs consisted of I/O (Input/Output), core, and memory.

According to the proposed architecture of a biomolecular computing system, the input is realized by translating the electrical input signal into biological stimuli, the information is deposited in a biomolecular computing core, and a biosensor transforms the information from the biological/biochemical format back into an electrical signal. The transformed electrical signal produces a final output appropriate for the following blocks in the computing and communication chain. The authors concluded that despite the many obstacles during the construction of computing designs based on biomolecules, biosensors are the most natural input/output tools for biomolecular computing structures, which have shown their reliability, high power, and capabilities in other areas of application.

## 5. Conclusions

In this review, we surveyed various current developments in magnetite-based biosensors for POC diagnostics; the synthesis, surface coverage, and functionalization of MNPs; as well as some perspectives in molecular logic. MNPs are frequently used for various medical–biological applications due to the benefits of MNPs, such as the capacity to magnetically control their accumulation, their superparamagnetic behavior, easy production, rapid separation inside buffer solutions, and sensitivity for signal detection. Together, these features of MNPs make it possible to efficiently complete nucleic acid purification, pre-concentration, and separation while maintaining specificity during the virus detection procedure. When paired with a fluorescent or chemiluminescent probe in real-time detection systems, MNPs are crucial components. Researchers can attach a variety of groups to MNPs to boost their chemical functionality, constancy, wettability, and bonding adaptability for a diversity of applications because the morphology, functionalization, surface coating, and characteristics of MNPs are highly adaptable. Magnetite nanoparticles might fill a void in the creation of next-generation pharmaceuticals for the diagnosis and treatment of diseases. Multifunctional MNPs with bioinspired coatings may also represent a significant advancement in nanomedicine. There are still many challenges to overcome before MNPs can be implemented in clinics. We could outline the prospects within MNPs developments for smart technologies (molecular computers) in the investigation regularities of biologically mineralized magnetite with the goal of the development of the bioinspired large-scale synthesis of particles with controllable sizes and shapes with biocompatible coatings. This may usher in a new era of nanomedicine if we can more effectively understand the physical, chemical, and biological issues as well as the principles of property manipulation for MNPs.

During the global pandemic, the significance of POC devices is hard to overestimate, as they enable the rapid quantitative and/or qualitative identification of human viruses and other pathogen infections. POC diagnostics has the following advantages compared with laboratory diagnostics: it is patient-centered, does not require trained staff/laboratory/complex equipment, and can be carried out at any time at school, at home, in the office, at an airport, etc. Biosensors are the backbone for the manufacture of POC devices, and over the last few years, research regarding new smart biosensor development has expanded rapidly. Among a number of other biosensors, the main benefit of MNP biosensors is their low background signal, as biological samples usually do not have noticeable magnetic signals. In addition, MNPs are relatively inexpensive, stable, and easily functionalized for further applications. As MNPs are employed in biosensors that utilize optical sensing technology, they could be promising in the development of POC devices combined with smartphones, which could considerably increase their portability, accessibility, digitalization, and integration within medical databases. Future perspectives of POC device developments are the creation of new powerful databases for information storage, as well as algorithms of information processing which would use the principles of Artificial Intelligence. One more conclusion that can be made is that the combination of MNPs with proper coverage/functionalization (biomolecules, CDs, Au, etc.) leads to their synergistic effects and results in the more sensitive and accurate identification of target biological objects.

Recently, molecular logic has become an alternative to logic operations based on silicon, and MNPs are used for the creation of various logic applications because of their special features, such as their susceptibility to external magnetic fields. The utilization of MNPs with integrated biocomputing interfaces can significantly increase the effectiveness and precision of logic applications. To date, the following MNP-based logic gates have been reported in the literature: “OR”, “AND”, “XOR”, and “INHIBIT”. The construction of double-input logic gates, particularly the “INHIBIT” gate and “AND” gate, is the subject of several studies across many disciplines. Although there is very little research regarding multi-input and multidomain-valued logic gates, it can be enhanced by combining them with nanodevices. Meanwhile, nanomaterials, as potential modules for upcoming biomedical nanorobots, are used to actualize the principles of biomolecular computing. The potential for nanorobots to cure cancer and other challenging diseases look particularly alluring in terms of the in-cell computing systems capable of influencing cell behavior.

One of the newly challenged applications of MNP-based biosensors is building biomolecular computing systems, in which biosensors will act as natural output devices based on molecular logic gates. Such new biomolecule-based computers could ensure higher data density and increased processing speed compared with existing computer architectures. As DNA becomes a benchmark in biomolecular computing, the future perspective of MNP development in this research field could be the creation and utilization of MNPs/DNA bioconjugates for the fabrication of building blocks of novel systems of information saving and processing.

We could conclude that progress in the knowledge and development of MNP-based biosensors, which proceeds with the creation and building of POC devices and molecular logic gates, could lead scientists to new solutions in the design of novel cutting-edge systems of biomolecular computing, which would be based on new ideas and principles.

## Figures and Tables

**Table 1:** Comparative table of the limit of detection (LOD) of biological objects detected via MNPs with specified read-out modes.

Biological Object	LOD	Testing Time	Read-Out Mode
SARS-CoV-2	5.9 fmole	Seconds	Magnetic [133]
	10 ng/mL	10 min	Magnetic [134]
	5 ng/mL	10 min	Magnetic [134]
	50 pg/mL	<1 h	Electrochemical [135]
	10 pg/mL	<1 h	Electrochemical [135]
	Protein S 6.31 pM	60 min	Electrochemical [136]
	Protein N 5.27 pM	60 min	Electrochemical [136]
Human chorionic gonadotropin (hCG)	0.025–0.044 ng/mL	Minutes	Optical [137]
Streptococcus mutants	12 CFU/mL	15 min	Optical [138]
*Listeria monocytogenes*, *Campylobacter jejuni*, and *Staphylococcus aureus*	10 cells/mL	<35 min	Optical [139]
*Escherichia coli*	50 cells/mL	15 min	Optical [140]
*Staphylococcus aureus*	20 cells/mL	15 min	Optical [140]
*Vibrio parahaemolyticus*	14 CFU/mL	-	Optical [141]
*Acinetobacter baumannii*	10^2^ CFU/reaction	30 min	Optical [142]
H1N1 virus	0.032 hemagglutination units/reaction	40 min	Optical [143]

## Data Availability

The data presented in this review are available from the original publications that are cited.
